# Physical fitness and blood parameters outcomes of breast cancer survivor in a low-intensity circuit resistance exercise program

**DOI:** 10.1515/med-2024-1010

**Published:** 2024-08-13

**Authors:** Keun-Ok An, Kwang-Jin Lee

**Affiliations:** Sports Medicine Major, Division of Sports, Korea National University of Transportation, Chungju, 27469, Korea; Department of Physical Education, Chungbuk National University, Cheongju, 28644, Korea

**Keywords:** physical fitness, circuit resistance training, natural killer cell activation, insulin-like growth factor binding protein 3, breast cancer survivors

## Abstract

There is limited evidence regarding the effect of circuit-type low-intensity resistance exercise on physical fitness and blood parameters in breast cancer survivors (BCSs). Therefore, this study aimed to investigate the effect of low-intensity circuit resistance exercise on changes in physical fitness and blood parameters in BCSs. A total of 16 BCSs participated in a low-intensity circuit resistance exercise group (LCREG). The exercise program in the LCREG consisted of 50–60% of one repetition maximum, two to three times weekly, for 24 weeks. The control group (CG) did not receive any interventions. All participants were measured for physical fitness and blood parameters before and after the exercise intervention. The results showed that LCREG significantly improved body mass index (BMI) (*p* = 0.012), grip strength (*p* = 0.017), back strength (*p* = 0.042), plank (*p* = 0.036), balance (*p* = 0.030), low-density lipoproteins (LDL) (*p* = 0.050), total cholesterol (*p* = 0.017), and natural killer cell activity (NKCA) (*p* = 0.035) after exercise compared to before exercise. The LCREG also significantly improved BMI (*p* = 0.001), grip strength (*p* = 0.014), plank (*p* = 0.018), balance (*p* = 0.012), LDL (*p* = 0.024), total cholesterol (*p* = 0.012), and NKCA (*p* = 0.036) compared to the CG. These findings suggest that low-intensity circuit resistance exercise can increase physical fitness levels and improve the blood index in BCSs.

## 1 Introduction

Physical activity decreases after a breast cancer diagnosis, and low physical function is observed for more than 5 years after breast cancer treatment [1,2]. Irwin et al. [3] reported a decrease in physical activity levels in 25% of patients with breast cancer, and 35% reported no change in physical activity levels during a 6-year follow-up period. A decrease in physical function leads to fatigue, sleep disturbances, pain, depression, and anxiety, contributing to a decline in the quality of life of breast cancer survivors (BCS) [4,5]. Additionally, in breast cancer patients, the treatment process may have negative effects on sexual function, such as vulvo-vaginal atrophy, potentially leading to outcomes related to reduced quality of life [6,7]. Therefore, effective intervention methods should be applied to improve the BCS’s physical and psychological conditions. Exercise is one of the most effective strategies for improving physical and psychological problems associated with BCS [8,9,10,11]. In a prior study, Schwartz et al. [12] classified 66 BCSs into an aerobic exercise group, a resistance exercise group, and a control group (CG), and observed their progress over 24 weeks. This analysis showed that cardiorespiratory function and muscle strength improved in both the aerobic exercise and resistance exercise groups. Burnham and Wilcox [13] reported that 10 weeks of aerobic exercise improved physical functions, such as cardiorespiratory function, and psychological problems in breast and rectal cancer survivors. In addition, Serra et al. [14] reported that resistance exercise positively affected muscle strength and blood parameters in BCS. As a result, exercise is a key factor in improving physical and psychological problems and recovering, maintaining, and improving the physical function of BCS.

In previous studies related to BCS, various exercise methods and strengths have been applied. Short-term moderate- and high-intensity exercise [10,15,16,17], and long-term low- and medium-intensity exercise [18,19,20,21] have been applied. In particular, high-intensity resistance exercise has been shown to bring greater health benefits [10,17]. However, because high-intensity exercise requires extreme energy consumption, a high level of motivation is required, which can lead to giving up or making it difficult to continue [22,23]. In addition, exercise should focus on restoring, maintaining, and enhancing BCS’s bodily functions safely and efficiently from a mid-to-long-term perspective rather than a short-term perspective. This suggests that it is necessary for BCS to increase resistance exercise adaptation by performing low rather than high-intensity resistance exercise. This is because the survival period and rate of BCS are increasing, and strategies to sustain a healthy life are required. Therefore, it is necessary to comprehensively review exercise type, intensity, duration, and interest to induce continuous participation and interest.

Circuit resistance exercise is effective in arousing interest and increasing the participation rate compared to other exercises because the program can be configured in various ways and is an exercise method that attracts attention from women with low physical fitness levels [24,25]. The dropout rate has been reported to be relatively low [22,26], so it is judged to be the most suitable form of exercise for BCS to participate continuously.

We hypothesized that low-intensity circuit resistance training increases physical fitness in BCSs. Further, we proposed that low-intensity circuit resistance exercise improves blood parameters in BCSs. Therefore, this study aimed to examine the effects of long-term low-intensity circuit resistance exercise on physical fitness and blood parameters in BCS.

## 2 Methods

### 2.1 Participants

**Figure 1:**
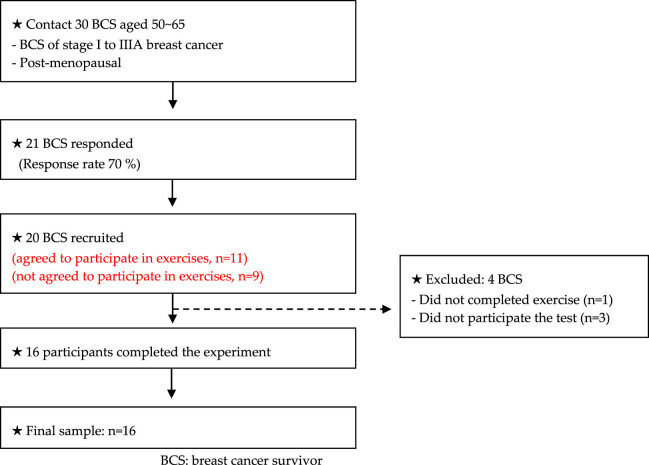
Flow diagram explaining criteria for participant selection.

Sixteen BCS participated in this study. The criteria for participation in the study were as follows: (a) Diagnosis of breast cancer stage: I–Ⅲ A, (b) 50–65 years of age, (c) menopause diagnosis (≥50 years of age), (d) no resistance exercise for 1 year, (e) completely cured breast cancer, ≥2 years, and (f) no musculoskeletal symptoms and ≥6 months. Subjects who agreed to participate in exercises were assigned to low-intensity circuit resistance exercise group (LCREG), and subjects who did not agree to participate in exercises were assigned to CG. All participants were assigned to either an LCREG; (*n* = 8, age: 55.1 ± 4.6, weight: 66.5 ± 6.4, and height: 1.58 ± 0.0) or a CG; (*n* = 8, age: 57.8 ± 2.9, weight: 63.5 ± 7.0, and height: 1.56 ± 0.0). All participants signed a consent form identical to the content approved by the Institutional Review Board (U1IRB2021-08). Our study was registered with the Clinical Research Information Service (https://cris.nih.go.kr; registration number: KCT0008261). The criteria for selecting subjects are shown in Figure 1.

### 2.2 study design

This study was a non-randomized clinical trial. Measurements were ongoing at Center A in Region C, starting at 8:00 AM and ending at 12:00 PM. The participants were measured for 2 days. On the first day of measurement, body composition, muscle strength, muscle endurance, and balance were measured. Light jogging and stretching for 10 min were performed before the measurements. All participants were prohibited from eating for 2 h, and exercise was prohibited for 24 h before measurement. Blood was collected on the second measurement day, and fasting was performed 12 h before blood collection. After 24 weeks of exercise intervention, post-measurement was conducted simultaneously by the same measurer and measurement method.

### 2.3 Measurement of physical fitness

The participants’ body composition (height, weight, body mass index [BMI]) was measured using Bioelectrical Impedance Analysis (InBody-770, InBody Co., Ltd., Seoul, South Korea). Muscle strength was measured using a grip strength machine (TKK-5401, Takei, Niigata, Japan) and a back muscle strength machine (TKK-5402, Takei, Niigata, Japan) [27]. This was measured twice, and the best value was used. Muscular endurance was measured using sit-ups [28] and plank tests [29]. The participants lie on a sit-up board, spread both feet by 30 cm, and bend the knees vertically. With both hands intertwined behind their head, he raises his upper body with the ‘start’ signal. As you lift your upper body, your back touches the ground again after your elbows touch your knee. This motion was repeated for 30 s. The plank position was performed in a push-up position with the elbow bent at 90° and the floor supported with the forearm. The pelvis and lumbar spine were maintained in a neutral posture, and the body was made in a straight line from the ankles to the knees, hips, pelvis, spine, and head. Balance was measured using the one-legged standing method with eyes closed. All measurements were conducted after one practice session before the measurements. During the measurement, the measurement was stopped and recorded if the hand fell on the pelvis or the lower extremity was not kept in a 90° posture. The measurements were performed twice, and the best record was used.

### 2.4 Measurement of blood sample measurement of physical fitness

Blood samples were collected before and after exercise. Ten milliliters blood samples were collected from a vein in the participants’s arm. All samples were collected between 8:00 am and 10:00 am. After blood collection, 1 ml of blood was stored in a dedicated tube (NK Vue; ATGen Co., Seongnam-si, Korea) for natural killer cell activation (NKCA) analysis. The remaining 9 ml blood samples were transported in a vacuum tube for high-density lipoprotein (HDL), low-density lipoprotein (LDL), total cholesterol, and IGFBP-3 (Insulin-like growth factor binding protein 3) analysis and then used for analysis. Immediately after blood collection, blood was centrifuged at 3,000 rpm for 5 min at room temperature to separate it from serum and stored frozen at −70°C. HDL, LDL, and total cholesterol were subjected to enzymatic colorimetric analysis using a Cobas 8000 C702 (Roche Diagnostics System, Basel, Switzerland). IGFBP-3 was subjected to chemiluminescent immunoassay using an IM MULITE 2000XPI (Siemens Healthineers, Forchheim, Germany). NKCA was subjected to immunoassays using an ELx808 reader (BioTek Instruments).

### 2.5 Exercise program

The low-intensity circuit resistance exercise (LCRE) consisted of eight upper and lower extremity exercises (leg press, seated row, leg extension, shoulder press, back extension, arm extension, hip adduction, and hip abduction). The LCRE for BCS was constructed by modifying and supplementing the circuit resistance exercise program used in the studies by Lee & An, Gauche et al., [25] and Hashemi et al. [30]. The 1 RM for each exercise was calculated using the Brzycki formula [31]. The participants performed at an exercise intensity of 40% based on 1 RM and set different exercise sets for each week (1–4 weeks; 3 sets, 5–8 weeks; 4 sets, 9–12 weeks; 5 sets). After the 12 weeks of exercise, it was performed at 50% intensity based on 1 RM, and the weekly exercise set (13–16 weeks, 3 sets; 17–20 weeks, 4 sets; 21–24 weeks, 5 sets) was set differently. The rest period between the sets was 3 min. All participants set the exercise intensity by remeasuring 1 RM once every 4 weeks. LCREG was conducted in the afternoon, and 60 min of exercise was performed 2–3 times a week. The LCRE program is presented in Table 1.

**Table 1:** Low-intensity circuit resistance training program

Weeks	Type	Set (rep)	Set rest	Intensity	Concentric and eccentric ratio	Time
1–4	– Leg press – Seated row – Leg extension – Shoulder press – Back extension – Arm extension – Hip adduction	3 (16)	3 min	40% (1 RM)	1:1	– Warm up: 10 min – Main exercise: 30–40 min – Cool down: 10 min
5–8		4 (16)				
9–12		5 (16)				
13–16		4 (14)		50% (1 RM)		
17–20		5 (15)				
20–24		6 (16)				

1 RM: one repetition maximum; rep: repetitions.

### 2.6 Statistical analysis

Data were analyzed using SPSS 23.0 (IBM Corp, Armonk, NY, USA). An independent samples *t*-test was performed to verify homogeneity between groups, and the results showed no differences between groups (*p* < 0.05). Nonparametric tests based on Shapiro–Wilk and Kolmogorov–Smirnov were performed. The two-tailed Mann–Whitney *U* test was used to examine differences between the groups. The two-tailed Wilcoxon test was used to determine the differences within the group. All significance levels were set at 0.05.


**Informed consent:** Informed consent was obtained from all individuals included in this study
**Ethical approval:** The study was conducted according to the guidelines of the Declaration of Helsinki and approved by the Institutional Review Board of U1 University (U1IRB2021-08).

## 3 Results

### 3.1 Changes in physical fitness

Changes in physical fitness before and after the intervention are shown in Table 2. BMI (*z* = 2.521, *p* = 0.012), grip strength (*z* = 2.380, *p* = 0.017), back strength (*z* = 2.033, *p* = 0.042), plank (*z* = 2.100, *p* = 0.036), and balance (*z* = 2.173, *p* = 0.030) of the LCREG showed significant within-group differences after the intervention compared with pre-intervention. The BMI (*z* = 2.546, *p* = 0.011) of the CG group showed significant differences after the intervention compared to pre-intervention. In the differences between the groups, there were significant differences in BMI (*z* = 3.361, *p* = 0.001), grip strength (*z* = 2.470, *p* = 0.014), plank (*z* = 2.365, *p* = 0.018), and balance (*z* = 2.522, *p* = 0.012).

**Table 2:** Changes in physical fitness

Variables				Within-group analysis		Between-group analysis (pre–post differences)	
		Pre	Post	*Z*	*p*-value	*Z*	*p*-value
		Mean (SD)	Mean (SD)				
BMI (kg/m^2^)	CG	20.30 (1.70)	20.60 (1.62)	2.546	0.011*	3.361	0.001**
	LCREG	21.03 (2.05)	20.15 (1.88)	2.521	0.012*		
Grip strength (kg)	CG	23.91 (4.70)	23.02 (3.64)	1.260	0.208	2.470	0.014*
	LCREG	24.80 (2.35)	27.22 (2.04)	2.380	0.017*		
Back strength (kg)	CG	49.26 (13.23)	48.70 (15.58)	0.420	0.674	1.524	0.128
	LCREG	52.71 (11.51)	54.72 (10.74)	2.033	0.042*		
Sit-up (rep)	CG	7.12 (4.38)	7.37 (5.47)	0.283	0.777	1.377	0.168
	LCREG	6.50 (5.09)	8.75 (4.97)	1.897	0.058		
Plank (s)	CG	28.18 (8.69)	27.01 (6.84)	1.014	0.310	2.365	0.018*
	LCREG	26.35 (7.36)	35.02 (8.51)	2.100	0.036*		
Balance (s)	CG	8.28 (4.41)	7.91 (3.21)	0.841	0.400	2.522	0.012*
	LCREG	7.28 (5.59)	11.55 (3.66)	2.173	0.030*		

Means ± SD: means and standard deviation; BMI: body mass index; LCREG: low-intensity circuit resistance exercise; group; CG: control group; *p ≤ 0.05, **p ≤ 0.01; rep: repetitions.

### 3.2 Changes in blood parameters

Changes in blood parameters before and after the intervention are shown in Table 3. LDL (*z* = 1.960, *p* = 0.050), total cholesterol (*z* = 2.380, *p* = 0.017), and NKCA (*z* = 2.103, *p* = 0.035) of the LCREG showed significant within-group differences after the intervention compared to pre-intervention. CG showed no significant differences after the intervention compared to pre-intervention. In the differences between the groups, there were significant differences in LDL (*z* = 2.263, *p* = 0.024), total cholesterol (*z* = 2.521, *p* = 0.012), and NKCA (*z* = 2.102, *p* = 0.036).

**Table 3:** Changes in blood parameters

Variable		Pre	Post	Within-group analysis		Between-group analysis (pre–post differences)	
				Z	*p*-value	*Z*	*p*-value
HDL (mg/dl)	CG	66.93 (18.61)	62.20 (16.87)	1.260	0.208	1.155	0.248
	LCREG	60.92 (21.52)	65.56 (17.52)	0.980	0.327		
LDL (mg/dl)	CG	126.25 (49.31)	132.50 (42.60)	1.825	0.050*	2.263	0.024*
	LCREG	118.75 (46.21)	98.37 (23.61)	1.960	0.050*		
Total cholesterol (mg/dl)	CG	214.87 (62.91)	226.75 (62.38)	1.332	0.183	2.521	0.012*
	LCREG	207.62 (58.22)	189.37 (47.88)	2.380	0.017*		
NKCA (pg/ml)	CG	641.87 (529.44)	630.75 (508.22)	0.560	0.575	2.102	0.036*
	LCREG	727.50 (876.31)	850.50 (837.26)	2.103	0.035*		
IGFBP-3 (μg/ml)	CG	3.73 (0.62)	3.74 (0.65)	0.700	0.484	1.575	0.115
	LCREG	4.29 (0.82)	4.17 (0.81)	1.521	0.128		

Means ± SD: means and standard deviation; HDL: high-density lipoprotein; LDL: low-density lipoprotein; NKCA: natural killer cell activation; IGFBP-3: insulin-like growth factor binding protein 3; LCRTG: low-intensity circuit resistance training group; CG: control group. *p ≤ 0.05.

## 4 Discussion

This study is the first to identify the effects of long-term LCRE on BCS. We hypothesized that LCREG would improve physical fitness and blood parameters compared with the CG. The results of applying LCRE twice a week for 24 weeks confirm our hypothesis that it improves fitness and blood parameters in BCS.

Cancer survivors are more likely to stop exercising after treatment and very rarely resume exercise after treatment [31]. Muscle strength loss frequently occurs during breast cancer treatment. Therefore, restoring or maintaining muscle strength is one of the most important activities for BCS [32]. Resistance exercise significantly improves body function, including body composition, physical fitness, and strength in BCS. Vizza et al. [6] reported that using Replen to treat dyspareunia during resistance training such as pelvic floor exercises can improve the quality of life of cancer patients by improving physical strength and sexual function. Cormi et al. [33] reported that high-intensity resistance exercise (1 RM 75–85%) for 12 weeks improved muscle strength in breast cancer patients with lymphedema. Lee and An also reported that eight weeks of high-intensity circuit resistance exercise applied to BCS positively improved muscle strength and body composition. In addition, moderate-intensity resistance exercise (1 RM 60–70%) was also found to positively affect muscle strength and body composition in BCS [34]. Despite these benefits, many BCS avoid upper-body activities, including resistance exercise [35]. Low fitness levels, fatigue, and upper body injuries have been cited as the causes. Considering these points, we identified for the first time the effect of LCRE that BCS can easily access. The results showed that LCREG positively significant differences in BMI, grip strength, back muscle strength, plank, and balance of BCS after the exercise intervention. In addition, LCREG was positively correlated with BMI, grip strength, plank, and balance to CG. Watanabe et al. [36] argued that low-intensity exercise with a long muscle contraction period effectively increased muscle strength without hypertrophy. Linero and Choi [37] reported that 12 weeks of low-intensity resistance exercise was a safe and effective way to improve strength and balance in postmenopausal women with osteopenia.

Breast cancer treatment increases body fat [38], blood lipids [38], IGFBP-3 [39], negative impact on sexual functioning such as vulvo-vaginal atrophy [6,7], and decreases the activation of natural killer cells. This symptom is persistent among BCS and contributes to reduced quality of life and mortality from breast cancer recurrence and complications [40]. High-intensity resistance exercise reduces IGFBP-1 and IGFBP-3, which are involved in cancer cell growth [41], reduces cholesterol [42], and enhances NKCA [17], which kills cancer cells. However, long-term high-intensity resistance exercise affects BCS reduced motivation to participate, physical and psychological burden, and occurrence of orthopedic problems [43]. Therefore, alternative methods of participating in resistance exercises while reducing these problems are needed. As an alternative method, we applied LCRE to BCS over a long period. LDL, total cholesterol, and NKCA levels in the LCREG showed positive changes after 24 weeks of exercise intervention. In addition, LCREG positively improved LDL, total cholesterol, and NKCA compared to CG. Although we did not confirm any studies that analyzed the effect of LCRE on blood markers in BCS, other studies have shown the effect of resistance training on these variables. Okamoto et al. [44] reported that low-intensity resistance exercise effectively reduced LDL and total cholesterol levels in healthy women, and home-based low-intensity resistance exercise positively improved NKCA in esophageal cancer survivors [45]. In particular, Hashemi et al. [30] reported that low-intensity (1 RM 40%) circuit resistance exercise effectively increased HDL, decreased LDL, and reduced triglycerides in postmenopausal women. It was found that 35% of circuit resistance exercises based on 1 RM positively affected the improvement of plasma retinol-binding protein-4 and tumor necrosis factor-α in postmenopausal women. The physiological mechanisms underlying the effects of LCRE on changes in blood markers can be explained by several factors. It should be noted that LCRE is effective in improving cholesterol levels, similar to aerobic exercise [30]. In addition, the production of mytokines induced by continuous muscle contraction may have positively improved blood marker levels [46]. Finally, it is possible that the set variables positively improved as exercise volume gradually increased [36]. Our findings are meaningful in promoting the health status of BCS and applying them as a method of exercise prescription. Of course, the evidence to claim that it is superior to high-intensity resistance exercise is very weak, but it is a viable alternative for BCS who are weak or hesitant to do high-intensity exercise. In particular, engaging in low-intensity circular exercise can increase adaptability to resistance exercise and increase health benefits. This may open up the possibility of applying high-intensity forms of resistance exercise in the future, and may provide more essential health benefits for BCS. However, the limitations of this study are as follows: (a) The small sample size. (b) The lack of comparison or comment on the benefit of low-intensity endurance exercise in breast cancer patients. (c) Different breast cancer treatment methods, diagnosis stages, and fitness levels. (d) Physical fitness, BMI, medication, and dietary interventions were not considered when recruiting participants. (e) This study was conducted as a pilot study.

## 5 Conclusion

Current research shows that long-term LCRE can promote physical activity and improve health-related parameters in BCS. Based on these findings, it would be practical to apply different exercise intensities, including low-intensity circuit resistance exercise, to more BCSs and conduct larger community-based trials to evaluate their health-related benefits. Future studies should use randomized methods to classify study participants and consider the combined effects of other lifestyle interventions, including exercise and dietary modifications.
